# A Cuffless Blood Pressure Measurement Based on the Impedance Plethysmography Technique

**DOI:** 10.3390/s17051176

**Published:** 2017-05-21

**Authors:** Shing-Hong Liu, Da-Chuan Cheng, Chun-Hung Su

**Affiliations:** 1 Department of Computer Science and Information Engineering, Chaoyang University of Technology, Taichung 41349, Taiwan; shliu@cyut.edu.tw; 2 Department of Biomedical Imaging and Radiological Science, China Medical University, Taichung 40402, Taiwan; 3 Institute of Medicine, School of Medicine, Chung-Shan Medical University; Department of Internal Medicine, Chung-Shan Medical University Hospital, Taichung 40201, Taiwan

**Keywords:** cuffless blood pressure measurement, impedance plethysmography, pulse transit time

## Abstract

In the last decade, cuffless blood pressure measurement technology has been widely studied because it could be applied to a wearable apparatus. Electrocardiography (ECG), photo-plethysmography (PPG), and phonocardiography are always used to detect the pulse transit time (PTT) because the changed tendencies of the PTT and blood pressure have a negative relationship. In this study, the PPG signal was replaced by the impedance plethysmography (IPG) signal and was used to detect the PTT. The placement and direction of the electrode array for the IPG measurement were discussed. Then, we designed an IPG ring that could measure an accurate IPG signal. Twenty healthy subjects participated in this study. The changes in blood pressure after exercise were evaluated through the changes of the PTT. The results showed that the change of the systolic pressure had a better relationship with the change of the PTT_IPG_ than that of the PTT_PPG_ (*r* = 0.700 vs. *r* = 0.450). Moreover, the IPG ring with spot electrodes would be more suitable to develop with the wearable cuffless blood pressure monitor than the PPG sensor.

## 1. Introduction

Some wearable techniques for healthcare devices have been developed over the last decade [1]. There are fingertip pulse oximeters to measure oxygen saturation (SpO_2_) [2], electrocardiography (ECG) [3,4], and devices to measure body temperature [5], physical activity [6,7], heart rate [8,9] and respiratory rate [10]. Some wearable devices with many sensors could measure a few physiological parameters; an ECG, for example, can monitor body temperature, heart rate, motion activity, etc., [11,12]. Nowadays, these wearable devices can be worn on the chest, wrist, waist, or ankle. Moreover, they could be made as shirts or vests and be worn on the body.

Most commercial, automatic blood pressure monitors primarily apply either the auscultatory or the oscillometric methods [13,14]. Both of these methods utilize an occlusive cuff as an external pressure source, wrapping around a subject’s upper arm to disclose the systolic and diastolic pressures within 30–60 s. Since the blood pressure monitor using the auscultatory or oscillometric method must include a cuff and some mechanical devices such as a pumping motor and a deflating valve, it could not be designed as a wearable apparatus. However, blood pressure is one of the most important physiological parameters. In addition, according to The World Health Organization’s report, people need to measure their blood pressure once a day. This could avoid blood pressure spikes, and thus reduce the risk of cardiovascular diseases [15].

Ahlstrom et al. proposed a novel technique for cuffless blood pressure measurement; they used the pulse transit time (PTT) to evaluate the systolic blood pressure [16]. They found they could monitor the changed tendency of a person’s blood pressure by using this method. Sharwood-Smith et al. used the PTT to evaluate the change of blood pressure during obstetric spinal anaesthesia [17]. The PTT is defined as the time from the systolic ending of the left ventricular to the specific position of the peripheral artery. Therefore, the cuffless technique must include at least two sensors, one placed on the chest and another one placed on the arm or the leg. Some studies used the ECG and photo-plethysmography (PPG) technique to measure the PTT [16,17]. Foo et al. [18], Chandrasekaran et al. [19], and Peng et al. [20] used phonocardiography to replace the ECG signal. Park et al. used the tonometer to replace the PPG to evaluate the blood pressure [21].

The impedance plethysmography (IPG) technique is a physical measure of the ionic conduction of a specific body segment in contrast with electrical conduction characteristics [22,23,24]. The transient and static values of electrical conductivity depend on the dynamic and balanced conditions of arteriovenous blood volume within a body segment. Thus, it could be used to measure the hemodynamic parameters, such as blood flow, stroke volume, and thoracic fluid. Impedance-cardiography is the most important application [25,26]. However, the most significant challenge for the IPG technique is the optimal placement of the electrode array, because the current distribution will be affected by body tissue and muscle [27]. The other problem is how to extract the IPG signal of the blood flow because the resistance of the pulse volume in a segment of an arm is usually from 0.05 to 0.1 ohm [22]. Moreover, body motions will make the baseline of the IPG signal vary. [24].

In this study, the goal was to develop an IPG sensor that could replace the PPG sensor to measure the arterial pulse. In order to find the optimal position and direction of the electrode array for the IPG sensor, we discovered many different current distributions. A drive circuit for the IPG sensor was designed which had a good signal-to-noise ratio. In total, 20 healthy subjects participated in this study, and their blood pressure was measured before and after doing exercise. We found that the relationship between the PTT and blood pressure was a polynomial function.

## 2. Materials and Methods

### 2.1. Impedance Plethysmography Technique

In a segment of the arm, *R_b_* is the total resistance that includes the statistic resistance, *R_o_*, and the alternate resistance for the blood flow pulse, *R_n_*,

(1) $$R_{b}=\frac{R_{o}R_{n}}{R_{o}-R_{n}}=\frac{R_{o}R_{n}}{\Delta R}$$

where *ΔR* is the changed resistance incurred by the change of blood volume in the segment [28]. The sensing IPG voltage, *V_IPG_*, is defined as

(2) $$V_{IPG}=I_{A}R_{b}$$

where *I_A_* is the input current. Thus, if *I_A_* is a constant current, *V_IPG_* and *R_b_* have a proportional relation.

**Figure 1:**
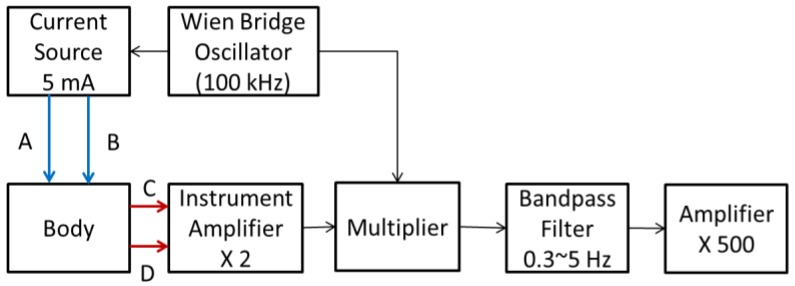
The block diagram of the drive circuit for the impedance plethysmography (IPG) measurement.

Figure 1 shows the block diagram of the drive circuit for the IPG measurement. A Wien bridge oscillator was used to generate the 100 kHz sinusoidal wave, which drove a constant current source, 5 mA. This radial frequency signal was input to the body by two transmitting electrodes (T1 and T2). An instrument amplifier (AD8422) was used to pick up the modulated signal by two receiving electrodes (R1 and R2). The demodulator was composed of a multiplier (AD9837) and a two-order low pass filter (cutoff frequency: 5 Hz), which extracted the IPG signal. The gain was 500, the cutoff frequency of the four-order low pass filters was 5 Hz to reduce high frequency noise, and the cutoff frequency of the one-order high pass filter was 0.3 Hz to reduce the wounding baseline. The operation amplifier was ADTL082.

**Figure 2:**
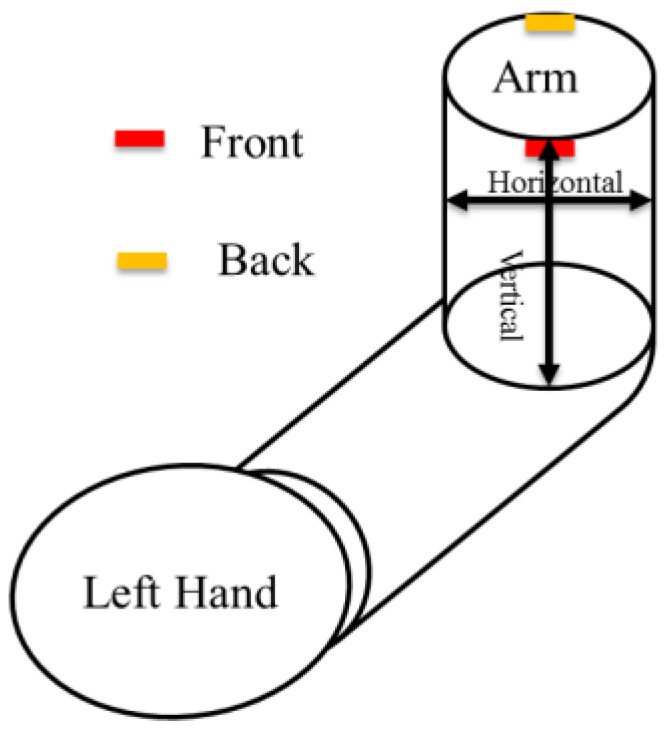
The definition for the horizontal and vertical direction on the arm. Red color represents the front side, the yellow-brown color represents the back side.

**Figure 3:**
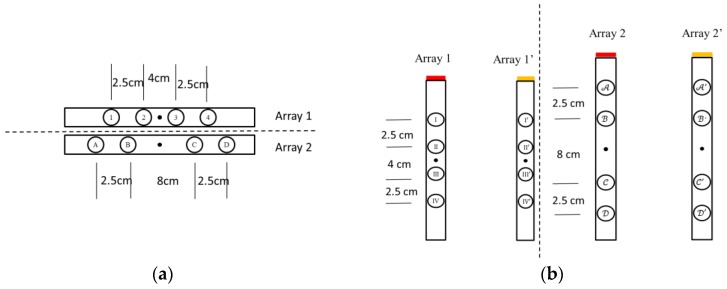
Two different sizes for the electrode array in two directions. (**a**) There are four electrodes in one array in the horizontal direction; (**b**) There are eight electrodes in one array in the vertical direction. Four electrodes are on the front side. The other four electrodes are on the back side.

The IPG technique for measuring arterial blood flow is based upon changes of the electrical impedance of blood caused by a cardio stroke. The impedance signal measured between two received electrodes is a voltage signal depending on the current distribution in a body segment between two transmitted electrodes. The signal-to-noise ratio of the IPG is related to the placed position and direction of the electrode array. Therefore, we studied several different positions and directions of the electrode arrays. The arm can be considered to be a cylinder. The electrode array was placed in a vertical and horizontal direction as shown in Figure 2. There are four electrodes in one array. The interval space of the electrode array is 2.5 cm and the center space is 4 cm or 8 cm. Figure 3a shows the electrode arrays of the different sizes, array 1 and array 2, in a horizontal direction. Figure 3b shows the electrode arrays of the different sizes, array 1 and array 2, in a vertical direction. Moreover, we also studied the mixed placement for the electrode array. Thus, there are two arrays in the vertical direction for the different sizes. One array with the red color is placed at the front. The other array with the yellow-brown color is placed at the back of the arm.

### 2.2. Impedance Plethysmography Signal

**Figure 4:**
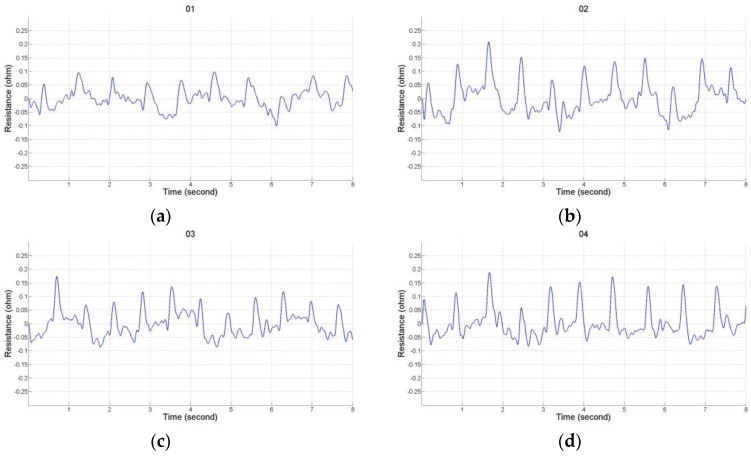
The electrode array is in the horizontal direction. (**a**) T_1_, T_2_ are 1 and 4, R_1_, R_2_ are 3 and 2; (**b**) T_1_, T_2_ are 1 and 3, R_1_, R_2_ are 4 and 2; (**c**) T_1_, T_2_ are A and D, R_1_, R_2_ are C and B; (**d**) T_1_, T_2_ are A and C, R_1_, R_2_ are D and B.

**Figure 5:**
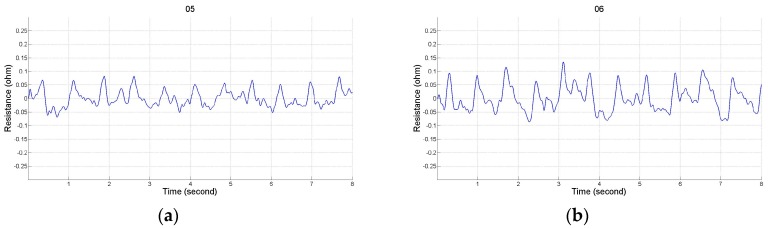

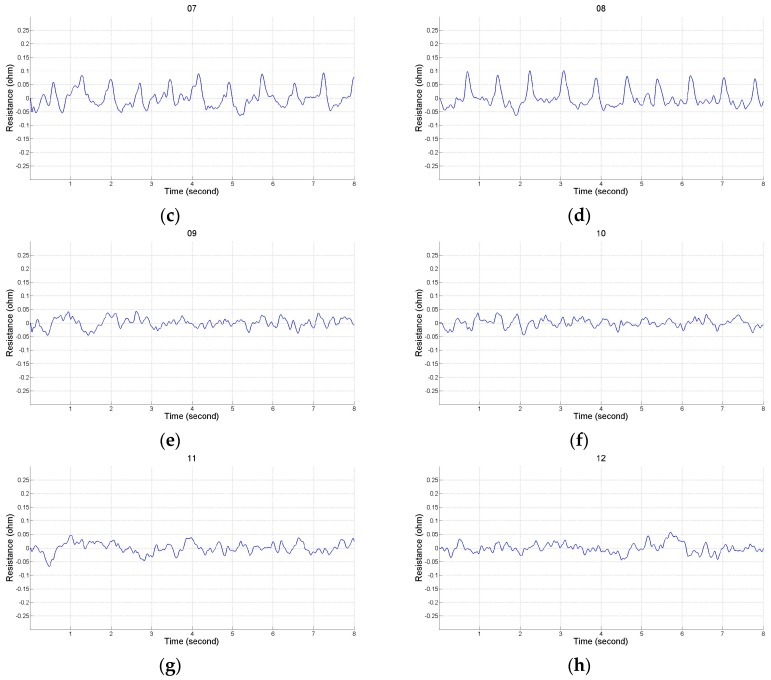
The electrode array is in the vertical direction. (**a**) T_1_, T_2_ are IV and I, R_1_, R_2_ are II and III; (**b**) T_1_, T_2_ are IV and II, R_1_, R_2_ are I and III; (**c**) T_1_, T_2_ are D and A, R_1_, R_2_ are ℬ and C; (**d**) T_1_, T_2_ are D and ℬ, R_1_, R_2_ are A and C; (**e**) T_1_, T_2_ are IV and II, R_1_, R_2_ are II’ and III’; (**f**) T_1_, T_2_ are IV and II, R_1_, R_2_ are I’ and III’; (**g**) T_1_, T_2_ are IV and I’, R_1_, R_2_ are II’ and III; (**h**) T_1_, T_2_ are IV and II’, R_1_, R_2_ are I’ and III.

**Figure 6:**
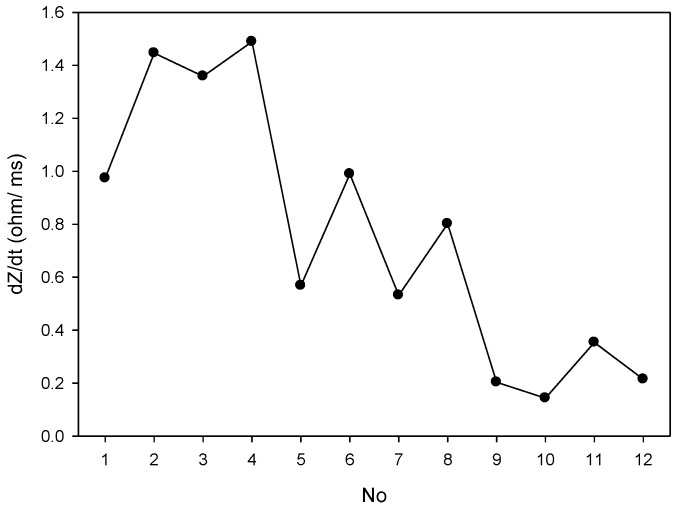
The change of ΔZ/Δt measurement from No. 1 to 12.

**Figure 7:**
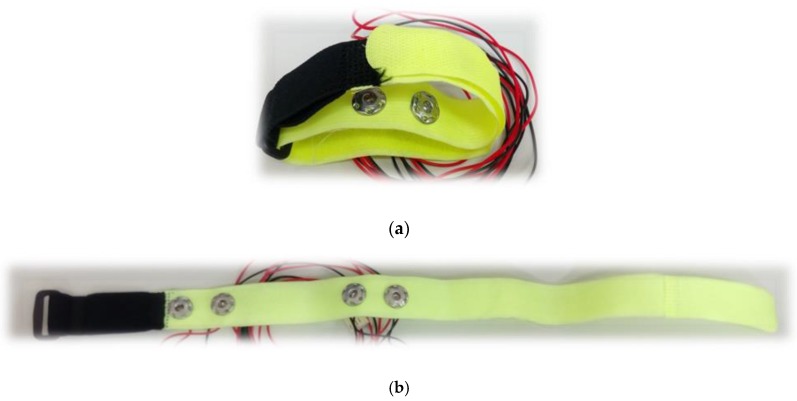
The real photo of the IPG ring with a width of 3 cm, and the bipolar electrode was in a symmetrical placement for the transmitting electrodes and receiving electrodes, (**a**) The IPG ring; (**b**) The extension of the IPG ring.

There were 12 placements for the electrode array as shown in Table 1. Numbers 1 to 4 belong to the placement of electrode array in a horizontal direction, and numbers 5 to 8 belong to the placement of electrode array in a vertical direction. Numbers 9 to 12 belong to the types of the mixed electrode array that have two electrodes on the front and back sides. The receiving electrodes are between the transmitting electrodes for the odd numbers, and the receiving electrodes and transmitting electrodes are alternately arranged for the even numbers. Figure 4 shows the IPG signals for one male subject. The electrode array was placed in a horizontal direction from numbers 1 to 4. Figure 5 shows the IPG signal for the same one in Figure 4. The electrode array was placed in a vertical direction from numbers 5 to 12. We found that significant pulses of blood flow could be found in Figure 4a–d and Figure 5a–d. However, the IPG signals in Figure 5e–h could not show clearer pulse signals than in Figure 5a–d. That is, the placements of the electrodes could not mix the vertical and horizontal directions at the same time. Then, the amplitude of ΔZ/Δt was used to evaluate the performance of the electrode arrays (shown in Figure 6). We found that the electrode array placed in a horizontal direction could obtain the best signal-to-noise ratio. Furthermore, the bipolar electrodes must be placed symmetrically for the transmitting electrodes and receiving electrodes. According to the electrode array for number 4, we designed an IPG ring with a width of 3 cm, and the Ag–AgCl spot electrode was used. Figure 7a shows the real photo of the IPG ring, and Figure 7b shows the extension of the IPG ring.

## 3. Cuffless Blood Pressure Measurement

A multi-channel physiological signal measurement system (KL-710, K & H MFG. CO. LTD., Taipei, Taiwan) was used to measure the ECG, PPG and IPG signals of which the sampling rate was 500 Hz. Twenty healthy students (10 men and 10 women) participated in this study, aged 21.8 ± 1.9 years (mean ± SD; range: 20–25 years), weight 60.9 ± 10.4 kg, height 167.1 ± 9.4 cm, and they did not have any cardiac diseases or injured limbs. This experiment was approved by the Research Ethics Committee of China Medical University & Hospital (No. CRREC-105-072).

### 3.1. Experimental Protocol

**Figure 8:**
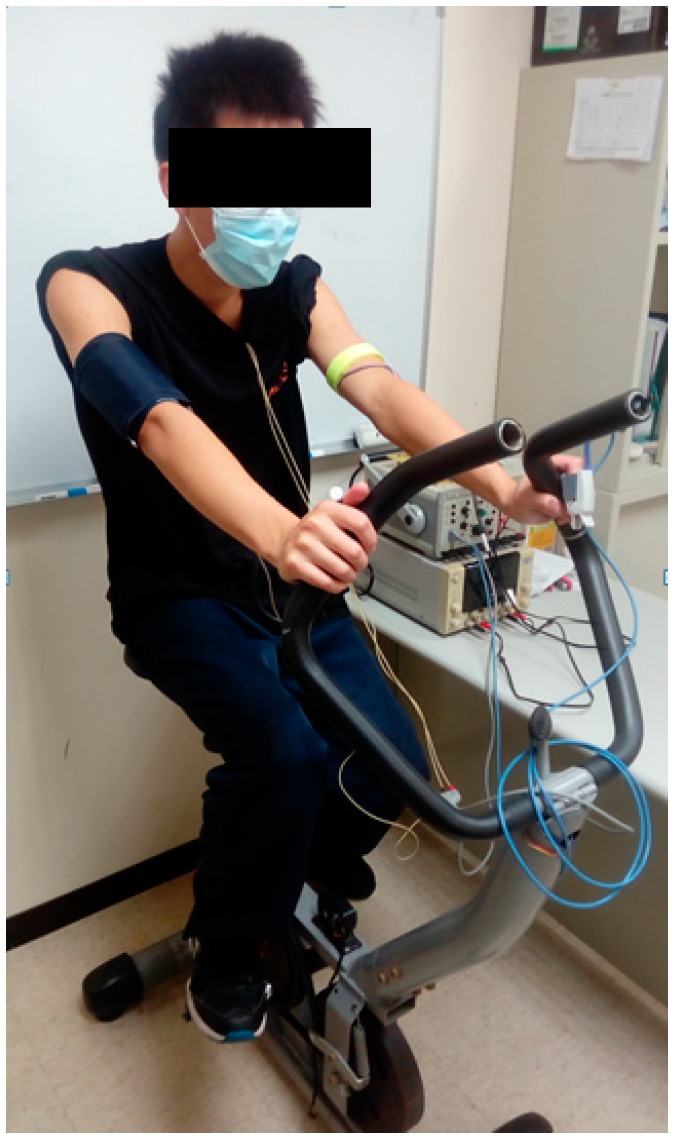
The real photo of the experiment.

An electronic blood pressure monitor (Omron, HM-7210, Japan) was used to measure their blood pressure, which was used as the accurate blood pressure in this experiment. The blood pressure monitor cuff was tied onto the right arm, the probe of the PPG was placed on the first finger of the left hand, and the IPG ring was placed on the left arm. The electrodes of the ECG were placed on the chest to measure the lead II signal. The subject rode on a fitness bike. Figure 8 shows the real photo of the experiment.

**Figure 9:**
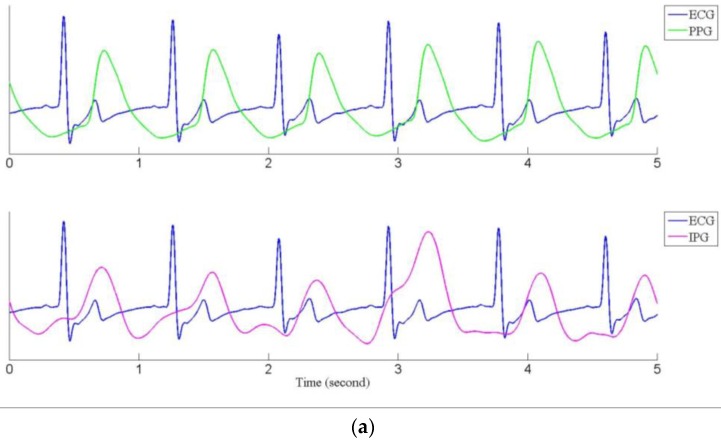

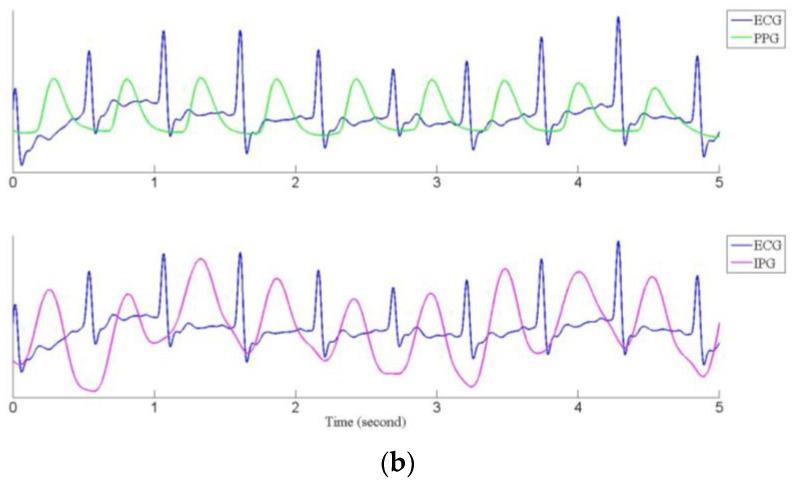
The ECG (blue), PPG (green) and IPG (purple) signals, (**a**) before exercise; (**b**) after exercise.

Subjects rested on the fitness bike for about five minutes, then their blood pressure was measured as the baseline. At the same time, the ECG, the PPG, and the IPG signals were recorded for 40 s. Subjects were requested to ride the fitness bike as intensely as possible for about 20 min. Their blood pressure was measured again. If their blood pressure was not higher than 20 mmHg for the baseline blood pressure, they had to ride the fitness bike continuously until their blood pressure measured higher than 20 mmHg. When measuring their blood pressure, the ECG, the PPG, and the IPG signals were measured again for 40 s. Then, the subjects would rest for about 10 min, and were requested to do the experiment again. Figure 9a shows the recorded ECG (blue line) and PPG (green line) signals, and Figure 9b shows the ECG and IPG (purple line) signals before and after riding the fitness bike. We found that the IPG signals were not more stable than the PPG signals. However, the main peaks of the PPG and the IPG were clear and obvious. The main peaks for the IPG signal were sharper than the PPG signal before exercise, especially in Figure 9a.

### 3.2. Pulse Transmission Time

The R peaks for the ECG, and the main peaks for the PPG and the IPG signals were marked by manual detection. The PTT_PPG_ is the PTT from the ECG and the PPG signals, and the PTT_IPG_ is from the ECG and the IPG signals. The PTT_PPG_ and PTT_IPG_ values were all determined by the means of the interquartile range. Table 2 shows the blood pressure, heart rate (HR), PTT_PPG_ and PPT_IPG_ parameters in the first test (before, systolic pressure (Sys): 112.9 ± 9.6 mmHg, diastolic pressure (Dia): 64.8 ± 6.7 mmHg, HR: 79.5 ± 10.5 beats per minute (BPM), PTT_PPG_: 315.5 ± 25.0 ms, PTT_IPG_: 293.6 ± 27.4 ms; after, Sys: 146.6 ± 9.6 mmHg, Dia: 63.8 ± 7.3 mmHg, HR: 110.8 ± 18.4 BPM, PTT_PPG_: 254.7 ± 24.9 ms, PTT_IPG_: 235.6 ± 16.1 ms), and Table 3 shows these parameters in the second test (before, Sys: 114.4 ± 10.2 mmHg, Dia: 70.6 ± 5.0 mmHg, HR: 86.6 ± 14.3 BPM, PTT_PPG_: 310.0 ± 19.9 ms, PTT_IPG_: 280.5 ± 23.8 ms; after, Sys: 145.2 ± 13.2 mmHg, Dia: 69.5 ± 8.4 mmHg, HR: 115.5 ± 21.4 BPM, PTT_PPG_: 252.9 ± 21.5 ms, PTT_IPG_: 219.2 ± 20.3 ms). We found that all parameters have a significant difference before and after exercise (*p* < 0.005) except the diastolic pressure in the first and second tests.

**Figure 10:**
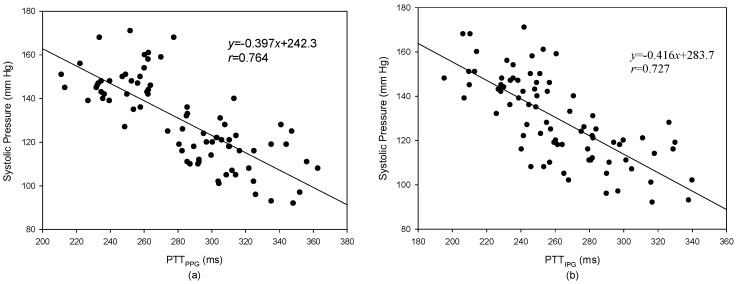
The result of the PTT_PPG_ and systolic pressure. (**a**) The relation between the PTT_PPG_ and systolic pressure, *r* = 0.764; (**b**) The relation between the PTT_IPG_ and systolic pressure, *r* = 0.727.

**Figure 11:**
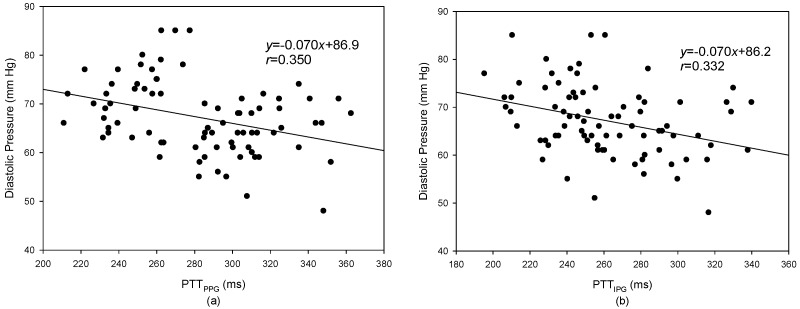
The result of the PTT_PPG_ and diastolic pressure. (**a**) The relationship between the PTT_PPG_ and diastolic pressure, *r* = 0.350; (**b**) The relationship between the PTT_IPG_ and diastolic pressure, *r* = 0.332.

**Figure 12:**
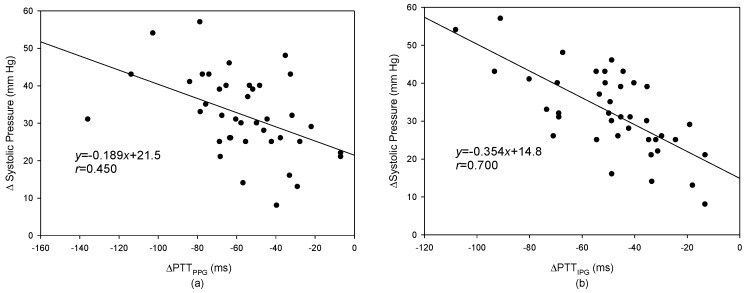
The relationship between the changes of (**a**) the PTT_PPG_ and the changes of systolic pressure, *r* = 0.450; (**b**) the PTT_IPG_ and the changes of systolic pressure, *r* = 0.700.

**Figure 13:**
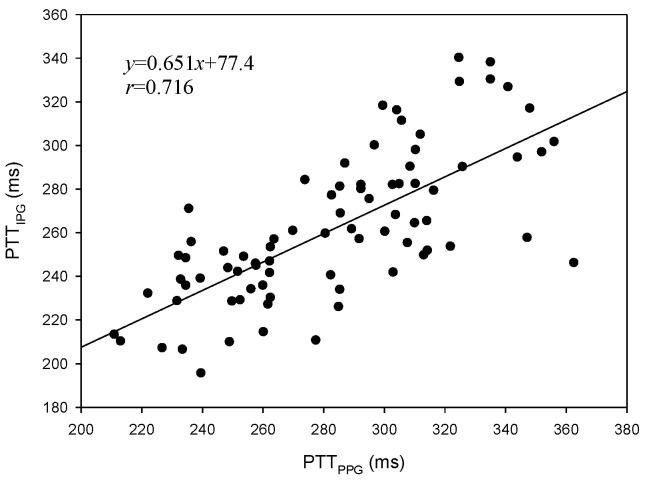
The correlation between PTT_PPG_ and PTT_IPG_, *r* = 0.716.

The levels of systolic pressure measured after exercise were significantly higher than before exercise. A linear regression was used to find the relation between the PTT and blood pressure. Figure 10a shows the result of the PTT_PPG_ and systolic pressure, the correlation coefficient is 0.764; Figure 10b shows the result of the PTT_IPG_ and systolic pressure, the correlation coefficient, *r*, is 0.727. Figure 11a shows the result of the PTT_PPG_ and diastolic pressure, the correlation coefficient is 0.350; Figure 11b shows the result of the PTT_IPG_ and diastolic pressure, the correlation coefficient is 0.332. The results for the change of systolic pressure before and after exercise and the change of the PTT are shown in Figure 12. The correlation coefficient (*r* = 0.700) for PTT_IPG_ was higher than it was for the PTT_PPG_ (*r* = 0.450). Figure 13 shows the correlation between the PTT_PPG_ and the PTT_IPG_ (*r* = 0.716). Although the PPG and IPG all belong to the plethysmograph technique, this result represents that the IPG signal is more sensitive to a change in the PTT than the PPG signal.

## 4. Discussion

In this study, we studied the placement position and direction of the electrode array that could measure an optimal IPG signal. When the bipolar electrodes were symmetrically placed for the transmitting and receiving electrodes and the electrode array was placed in a horizontal direction, the measured IPG signal had a maximum signal-to-noise ratio. However, this placement is very different to the previous study [22], which suggested that the electrode array was placed in a vertical direction. There were more uniform potential gradients between the receiving electrodes. Moreover, they were placed where the isopotential lines did not converge. However, this result could be accepted because Nyboer’s study used band electrodes. Spot electrodes were used in this study, which is similar to the electric dipole. Thus, the distribution of the electric dipole potential lines would be uniform between the two spot electrodes.

The statistic resistance including skin, soft tissue, and muscle is much larger than the changed resistance of the blood flow pulse in this measured segment. Thus, when the direction of the electrode array is placed in a horizontal direction, the statistic resistance would be reduced. As such, the background noise for the IGP signal could be reduced as well. Moreover, when the electrode array was placed in a horizontal direction, the electric fields produced by the spot electrodes would be in a cross section. The impedance of this section was measured continuously.

In recent years, the PPG technique, using the reflection method to detect the blood plethysmography, has been widely used to detect the heart rate on some wearable apparatuses, such as a fitness ring or a watch [28,29]. Thus, the wearable apparatus must fit tightly onto the skin because the direction of the PPG sensor must be perpendicular to the surface of the body. However, the placement of the bipolar electrodes on our design of the IPG ring is in a symmetrical direction. Therefore, it has three more benefits than the PPG technique. First, the IPG ring could be rotated around the arm and can detect the pulse signal because the arm could be considered to be a cylinder, and the electrodes of the IPG ring were placed symmetrically. Second, the impedance measurement was only affected by the distribution of potential field lines in the area. Thus, the electrodes only need to make slight contact with the skin; the potential field could be produced. Third, the electrodes could be made from soft materials. The electrodes can fit tightly on the skin, and users would not feel uncomfortable.

In this study, the increase in blood pressure was through exercise. Thus, the systolic pressure could be increased easier than the diastolic pressure. However, when the exercise stopped, the blood pressure quickly dropped. From Figure 10 and Figure 11, the results for the relationship between the PTT and the systolic pressure are better than the results for the relationship between the PTT and the diastolic pressure. This is the same as the previous studies [18]. Although the PTT_PPG_ used to evaluate the systolic pressure was a little better than the PTT_IPG_ (*r* = 0.764 vs. *r* = 0.727), the change of the PTT_IPG_ used to evaluate the change of the systolic pressure was much better than the change of the PPT_PPG_ (*r* = 0.700 vs. *r* = 0.450). This result may represent the PTT detected by the IPG technique suited to evaluate the transient change of blood pressure.

In the clinical application, the changes of the PTT are interesting in a non-invasive beat-to-beat index of blood pressure changes. When the blood pressure is increased by an increase in arterial stiffness, the relationship is nonlinear at high and low pressures [23]. If the blood pressure is changed by more than 10 mmHg, the PTT accurately tracking the change would drop to 67%. In this study, the systolic pressures after exercise were all more than 20 mmHg. Thus, the results of our study were worse than those of the previous studies [16,17,18,19].

## 5. Conclusions

In this study, we discussed the placement and direction of the electrode array for the IPG measurement when spot electrodes were used. The electrode array was placed in a horizontal direction, and the bipolar electrodes for the transmitting and receiving electrodes were placed in symmetrical placements. The measured IPG signal had a better signal-to-noise ratio. Therefore, we designed an IPG ring that was worn on the arm. The drive circuit for the IPG ring was also designed to detect the changes of the blood volume by the blood flow pulse. Finally, the ΔPTT_IPG_ to evaluate the change of systolic pressure before and after exercise performed better than the ΔPTT_PPG_. Therefore, the IPG technique performed better than the PPG technique when used to track changes in blood pressure, and would be more suitable to develop as a wearable apparatus.

## Figures and Tables

**Table 1:** There were twelve placements for the electrode array according to Figure 3.

No.	Electrodes T_1_, T_2_	Electrodes R_1_, R_2_	No.	Electrodes T_1_, T_2_	Electrodes R_1_, R_2_
1	$\vec{14}$	$\vec{32}$	2	$\vec{13}$	$\vec{42}$
3	$\vec{\mathrm{AD}}$	$\vec{CB}$	4	$\vec{\mathrm{AC}}$	$\vec{\mathrm{DB}}$
5	$\vec{\mathrm{IV}\;I}$	$\vec{\mathrm{II}\;\mathrm{III}}$	6	$\vec{\mathrm{IV}\;\mathrm{II}}$	$\vec{I\;\mathrm{III}}$
7	$\vec{DA}$	$\vec{ℬC}$	8	$\vec{Dℬ}$	$\vec{AC}$
9	$\vec{\mathrm{IV}\;\mathrm{II}}$	$\bar{\mathrm{II}\prime \;\mathrm{III}\prime }$	10	$\vec{\mathrm{IV}\;\mathrm{II}}$	$\bar{I\prime \;\mathrm{III}\prime }$
11	$\vec{\mathrm{IV}\;I\prime }$	$\vec{\mathrm{II}\prime \mathrm{III}}$	12	$\vec{\mathrm{IV}\;\mathrm{II}\prime }$	$\vec{I\prime \;\mathrm{III}}$

**Table 2:** In the first test, the measurement parameters before and after exercise.

	Before Exercise					After Exercise				
Subject No.	Sys (mmHg)	Dia (mmHg)	HR (BPM)	PTT_PPG_ (ms)	PTT_IPG_ (ms)	Sys (mmHg)	Dia (mmHg)	HR (BPM)	PTT_PPG_ (ms)	PTT_IPG_ (ms)
1	108	61	84	300	318	145	66	109	232	249
2	121	68	79	314	252	135	69	81	286	234
3	108	55	74	292	257	126	57	82	285	226
4	124	60	73	306	311	142	58	112	264	257
5	108	63	81	312	305	146	78	124	237	256
6	92	62	67	335	338	137	73	100	258	245
7	114	71	76	335	330	156	82	110	270	261
8	105	61	72	325	340	146	67	127	222	232
9	113	62	107	274	284	135	50	141	211	213
10	111	61	73	317	279	136	53	111	256	234
11	108	65	82	356	301	162	79	126	278	210
12	108	60	94	287	292	142	64	128	233	238
13	107	65	72	363	246	136	67	131	227	207
14	124	61	69	283	277	145	77	102	250	228
15	112	61	87	311	298	155	76	102	262	247
16	123	65	75	289	262	144	60	122	262	227
17	112	70	79	344	294	164	79	130	260	214
18	91	48	64	348	317	140	64	72	313	250
19	115	62	93	310	282	137	75	107	254	249
20	113	66	89	309	290	153	69	100	235	236
Mean ± Std	112.9 ± 9.6	64.8 ± 6.7	79.5 ± 10.5	315.5 ± 25.0	293.6 ± 27.4	146.6 * ± 9.6	63.8 ± 7.3	110.8 * ± 18.4	254.7 * ± 24.9	235.6 * ± 16.1

Sys: systolic pressure, Dia: diastolic pressure, HR: heart rate, PTT: pulse transmission time. ps: * represent *p* < 0.005.

**Table 3:** In the second test, the measurement parameters before and after exercise.

	Before Exercise					After Exercise				
Subject No.	Sys (mmHg)	Dia (mmHg)	HR (BPM)	PTT_PPG_ (ms)	PTT_IPG_ (ms)	Sys (mmHg)	Dia (mmHg)	HR (BPM)	PTT_PPG_ (ms)	PTT_IPG_ (ms)
1	114	54	84	297	300	144	62	118	247	222
2	106	63	78	322	254	114	54	84	283	210
3	110	50	70	293	282	131	58	77	286	259
4	125	60	82	303	282	146	61	129	235	228
5	102	63	98	304	316	141	74	128	236	258
6	95	66	73	326	290	141	73	114	262	229
7	123	68	76	341	327	156	82	116	263	241
8	105	58	91	304	268	130	63	121	249	227
9	104	53	128	325	329	133	56	141	213	198
10	115	50	91	300	260	140	52	129	232	213
11	115	62	96	310	264	145	74	116	253	204
12	109	64	103	293	280	152	70	130	260	228
13	121	65	97	303	242	147	74	125	240	184
14	117	64	71	281	259	149	72	103	249	199
15	125	68	81	305	282	165	75	103	252	220
16	110	60	91	314	265	149	63	136	263	218
17	118	70	90	347	258	161	76	153	234	184
18	96	58	63	352	297	127	51	65	308	209
19	118	68	87	295	275	144	79	112	258	231
20	119	64	85	286	281	147	71	110	240	226
	114.4 ± 10.2	70.6 ± 5.0	86.6 ± 14.3	310.0 ± 19.9	280.5 ± 23.8	145.2 * ± 13.2	69.5 ± 8.4	115.5 * ± 21.4	252.9 * ± 21.5	219.2 * ± 20.3

ps: * represent *p* < 0.005.

## References

[1] Majumder S., Mondal T., Deen M.J. (2017). Wearable sensors for remote health monitoring. Sensors.

[2] Tremper K.K., Barker S.J. (1989). Pulse oximetry. Anesthesiology.

[3] Medtronic Cardiac Diagnostics & Monitoring for Healthcare Professionals, SEEQ MCT System. http://www.medtronicdiagnostics.com/us/cardiac-monitors/seeq-mct-system/index.htm.

[4] Clearbridge VitalSigns, About CardioLeaf ULTRA. http://www.clearbridgevitalsigns.com/ultra.html.

[5] Buller M.J., Tharion W.J., Hoyt R.W., Jenkins O.C. Estimation of human internal temperature from wearable physiological sensors. Proceedings of the 22nd Conference on Innovative Applications of Artificial Intelligence.

[6] Liu S.H., Cheng W.C. (2012). Fall detection with the support vector machine during scripted and continuous unscripted activities. Sensors.

[7] Liu S.H., Chang Y.J. (2009). Using accelerometers for physical actions recognition by a neural fuzzy network. Telemed. J. E Health.

[8] Liu S.H., Cheng D.C., Lin C.M. (2013). Arrhythmia identification with two-lead electrocardiograms using artificial neural networks and support vector machines for a portable ECG monitor system. Sensors.

[9] Park J.H., Jang D.G., Park J., Youm S.K. (2015). Wearable sensing of in-ear pressure for heart rate monitoring with a piezoelectric sensor. Sensors.

[10] Liu G.Z., Guo Y.W., Zhu Q.S., Huang B.Y., Wang L. (2011). Estimation of respiration rate from three-dimensional acceleration data based on body sensor network. Telemed. J. E Health.

[11] Chen W., Dols S., Oetomo S.B., Feijs L. Monitoring body temperature of newborn infants at neonatal intensive care units using wearable sensors. Proceedings of the 5th International Conference on Body Area Networks.

[12] Guo D., Tay F.E., Xu L., Yu L., Nyan M., Chong F., Yap K., Xu B. A long-term wearable vital signs monitoring system using BSN. Proceedings of the 11th Euromicro Conference on Digital System Design Architectures, Methods and Tools.

[13] Carr J.J., Brown J.M. (2001). Introduction to Biomedical Equipment Technology.

[14] Liu S.H., Lin C.T. (2001). A model-based fuzzy logic controller with Kalman filtering for tracking mean arterial pressure. IEEE Trans. Syst. Man Cybern. Part A Human Syst..

[15] NCD Risk Factor Collaboration (2017). Worldwide trends in blood pressure from 1975 to 2015: A pooled analysis of 1479 population-based measurement studies with 19·1 million participants. Lancet.

[16] Ahlstrom C., Johansson A., Uhlin F., Länne T., Ask P. (2005). Noninvasive investigation of blood pressure changes using the pulse wave transit time: a novel approach in the monitoring of hemodialysis patients. J. Artif. Organs.

[17] Sharwood-Smith G., Bruce J., Drummond G. (2006). Assessment of pulse transit time to indicate cardiovascular changes during obstetric spinal anaesthesia. Br. J. Anaesth..

[18] Foo J.Y.A., Lim C.S., Wang P. (2006). Evaluation of blood pressure changes using vascular transit time. Physiol. Meas..

[19] Chandrasekaran V., Dantu R., Jonnada S., Thiyagaraja S., Subbu K.P. (2013). Cuffless differential blood pressure estimation using smart phones. IEEE Trans. Biomed. Eng..

[20] Peng R.C., Yan W.R., Zhang N.L., Lin W.H., Zhou X.L., Zhang Y.T. (2015). Cuffless and continuous blood pressure estimation from the heart sound signals. Sensors.

[21] Park M., Kang H., Huh Y., Kim K.C. Cuffless and noninvasive measurement of systolic blood pressure, diastolic blood pressure, mean arterial pressure and pulse pressure using radial artery tonometry pressure sensor with concept of Korean traditional medicine. Proceedings of the 29th Annual International Conference of the IEEE Engineering in Medicine and Biology Society.

[22] Nyboer J. (1950). Electrical impedance plethysmography: A physical and physiologic approach to peripheral vascular study. Circulation.

[23] Yamakoshi K.I., Shimazu H., Togawa T., Fukuoka M., Ito H. (1980). Noninvasive measurement of hematocrit by electrical admittance plethysmography technique. IEEE Trans. Biomed. Eng..

[24] Sherwood A., Allen M.T., Fahrenberg J., Kelsey R.M., Lovallo W.R., Doornen L.J.P. (1990). Methodological guideline for impedance cardiography. Psychophysiology.

[25] Kubicek W.G., Karngis J.N., Patterson R.P., Witsoe D.A., Mattson R.H. (1966). Development and evaluation of an impedance cardiograph system. Aerosp. Med..

[26] Bernstein D.P. (1986). A new stroke volume equation for thoracic electrical bioimpedance: Theory and rationale. Crit. Care Med..

[27] Qu M., Zhang Y., Webster J.G., Tompkins W.J. (1986). Motion artifact from spot and band electrodes during impedance cardiography. IEEE Trans. Biomed. Eng..

[28] Garmin Vivofit 3 Review. https://www.wareable.com/garmin/garmin-vivofit-3-review.

[29] The Best Apple Watch Apps: 50 Apps Tried and Tested. https://www.wareable.com/apple-watch/best-apple-watch-apps-832.

